# The Feasibility of a Using a Smart Button Mobile Health System to Self-Track Medication Adherence and Deliver Tailored Short Message Service Text Message Feedback

**DOI:** 10.2196/13558

**Published:** 2019-06-25

**Authors:** Rebecca J Bartlett Ellis, James H Hill, K Denise Kerley, Arjun Sinha, Aaron Ganci, Cynthia L Russell

**Affiliations:** 1 Science of Nursing Care Department Indiana University School of Nursing Indianapolis, IN United States; 2 Department of Computer & Information Science Purdue School of Science Indiana University-Purdue University Indianapolis, IN United States; 3 Center for Software and Innovation Purdue School of Science Indiana University-Purdue University Indianapolis, IN United States; 4 Richard L Roudebush VA Medical Center Division of Nephrology Indiana University Indianapolis, IN United States; 5 Visual Communication Design Herron School of Art and Design Indiana University-Purdue University Indianapolis Indianapolis, IN United States; 6 School of Nursing and Health Studies University of Missouri-Kansas City Kansas City, MO United States

**Keywords:** medication adherence, medication compliance, behavior change

## Abstract

**Background:**

As many as 50% of people experience medication nonadherence, yet studies for detecting nonadherence and delivering real-time interventions to improve adherence are lacking. Mobile health (mHealth) technologies show promise to track and support medication adherence.

**Objective:**

The study aimed to evaluate the feasibility and acceptability of using an mHealth system for medication adherence tracking and intervention delivery. The mHealth system comprises a smart button device to self-track medication taking, a companion smartphone app, a computer algorithm used to determine adherence and then deliver a standard or tailored SMS (short message service) text message on the basis of timing of medication taking. Standard SMS text messages indicated that the smartphone app registered the button press, whereas tailored SMS text messages encouraged habit formation and systems thinking on the basis of the timing the medications were taken.

**Methods:**

A convenience sample of 5 adults with chronic kidney disease (CKD), who were prescribed antihypertensive medication, participated in a 52-day longitudinal study. The study was conducted in 3 phases, with a standard SMS text message sent in phases 1 (study days 1-14) and 3 (study days 46-52) and tailored SMS text messages sent during phase 2 (study days 15-45) in response to participant medication self-tracking. Medication adherence was measured using: (1) the smart button and (2) electronic medication monitoring caps. Concordance between these 2 methods was evaluated using percentage of measurements made on the same day and occurring within ±5 min of one another. Acceptability was evaluated using qualitative feedback from participants.

**Results:**

A total of 5 patients with CKD, stages 1-4, were enrolled in the study, with the majority being men (60%), white (80%), and Hispanic/Latino (40%) of middle age (52.6 years, SD 22.49; range 20-70). The mHealth system was successfully initiated in the clinic setting for all enrolled participants. Of the expected 260 data points, 36.5% (n=95) were recorded with the smart button and 76.2% (n=198) with electronic monitoring. Concordant events (n=94), in which events were recorded with both the smart button and electronic monitoring, occurred 47% of the time and 58% of these events occurred within ±5 min of one another. Participant comments suggested SMS text messages were encouraging.

**Conclusions:**

It was feasible to recruit participants in the clinic setting for an mHealth study, and our system was successfully initiated for all enrolled participants. The smart button is an innovative way to self-report adherence data, including date and timing of medication taking, which were not previously available from measures that rely on recall of adherence. Although the selected smart button had poor concordance with electronic monitoring caps, participants were willing to use it to self-track medication adherence, and they found the mHealth system acceptable to use in most cases.

## Introduction

### Background

An estimated 30% to 50% of people with chronic conditions do not take medications as prescribed (eg, miss or skip doses, take medications late, or not at all), known broadly as medication nonadherence [1,2]. People who miss or skip taking medications or take them late are at risk for stopping their medications altogether [3]. Across all health conditions, medication nonadherence contributes to prescription-related morbidity and mortality, and nonadherence is estimated to cost around US $528 billion annually [4]. Despite the potential to improve patient outcomes associated with nonadherence, developing effective interventions relies on measuring medication adherence behaviors in a way that provides actionable information for behavior change.

Medication adherence measurement methods vary widely and include patient self-report (eg, questionnaires, interviews, and diaries), pill counts and claims data, direct observation, laboratory testing and monitoring by electronic technologies [5] (eg, packaging devices, digital medicines, ie, ingestible sensors), and video monitoring. Self-report and use of electronic technologies are traditionally and more frequently reported methods for measuring adherence in studies to improve medication adherence, as they offer insight into medication-taking behaviors useful for intervention [5,6]. Evidence suggests that electronic monitoring is better at detecting poor adherence compared with self-report [7,8], which often relies on recall. However, self-report is low cost and relatively easy to implement [6]. An advantage of electronic monitors is the ability to compile details about dosing history, as electronic monitors provide the time and date the medications were taken.

Currently used technologies that electronically compile dosing histories to determine adherence include packaging devices, digital medicines [9-11] (ie, ingestible sensors), and video monitoring [12]. Electronic packaging devices of pill caps, pillboxes, and blister packs differ by manufacturer, but they broadly use sensors to detect state changes in devices, for example, sensing cap or lid openings or closings, which indicate that medication has been taken. These “taking” events are associated with a date and time stamp to determine timing adherence, and these events have evidence of reliability for medication adherence both in clinical and research settings [13]. Although packaging devices can detect taking and timing adherence, using them requires disruption of already established medication-taking routines and organization systems, as users must store their medications in these devices for tracking [14,15]. Newer technologies, such as the ingestible sensor or video monitoring, overcome limitations imposed by packaging devices. The ingestible sensor allows users to keep their established routines, but it requires users to wear an adhesive patch attached to the abdomen to sense pill ingestion. The comfort associated with wearing the patch produced mixed reviews by patients [9]. In addition, ingestible sensors and video monitoring are intrusive and may be more appropriate for medications that require direct supervision to determine if medication was actually taken [16,17].

### Specific Objectives

Capitalizing on the ease of self-report measures, allowing for end user flexibility with the methods already in use to manage medications and capitalizing on the dosing history that can be compiled through electronic technology, we investigated the feasibility of patients using a smart button to self-track medication adherence. The purpose of this study was to evaluate the feasibility of using a novel approach to measure medication adherence in a way that capitalizes on the ease of self-report and the ability to electronically compile dosing histories by having patients self-track medication taking using a smart button. The smart button is a component of our mobile health (mHealth) system that was field tested with the smart button in this study. In addition to describing our mHealth system in this paper, we (1) describe recruitment, enrollment and participant characteristics, (2) report the number of times we successfully set up the mHealth system in the clinic setting, (3) describe participants’ willingness to use the mHealth system and instances when they did not desire to use the system, and (4) evaluate concordance between self-report data acquired using the adherence self-tracking feature of our mHealth system compared with an established packaging device.

## Methods

### Study Design

In preparation for future studies, we conducted this small feasibility study in 3 phases over a 52-day period, with repeated daily measurements of medication adherence. Phase 1 lasted for 14 days, and it was designed to introduce participants to using the smart button to self-track medication taking and receiving standard short message service (SMS) text messages, whereas phase 2 comprised 30 days of tailored SMS text messages. Phase 3 lasted for 7 days, and participants again received the standard SMS text message when the button was pressed. As this was a feasibility study, the focus was on understanding whether the smart button technology could be used by patients in their home environments. Our goal was to obtain 260 data points while minimizing the participant burden in the chance that the smart button technology did not work. As a result, we consecutively enrolled 5 participants. Data were collected between March 2018 and June 2018. Institutional review board approval was obtained, and all participants provided written informed consent before beginning the study.

### Participants

Individuals aged 21 years of age or older, with a diagnosis of chronic kidney disease (CKD) and who self-administered at least one antihypertensive medication daily, were eligible for study participation. In addition, individuals needed to be able to speak, hear, and understand English, have the ability to open pill bottle caps, and be willing to use study devices. If participants were receiving dialysis at the time of screening, they were excluded because of the burden of dialysis treatment. Cognitive impairment was assessed, and only those with a score of 4 or greater on the 6-item Metal Status Screen Derived from the Mini-Mental Status Exam were included [18].

### Setting

Participants were recruited from an ambulatory nephrology and hypertension clinic within the Indiana University Health system in Indianapolis, Indiana. Nephrologists prescreened patients taking antihypertensive medications and referred them to the research assistant (RA) for further screening.

### Mobile Health System

**Figure 1:**
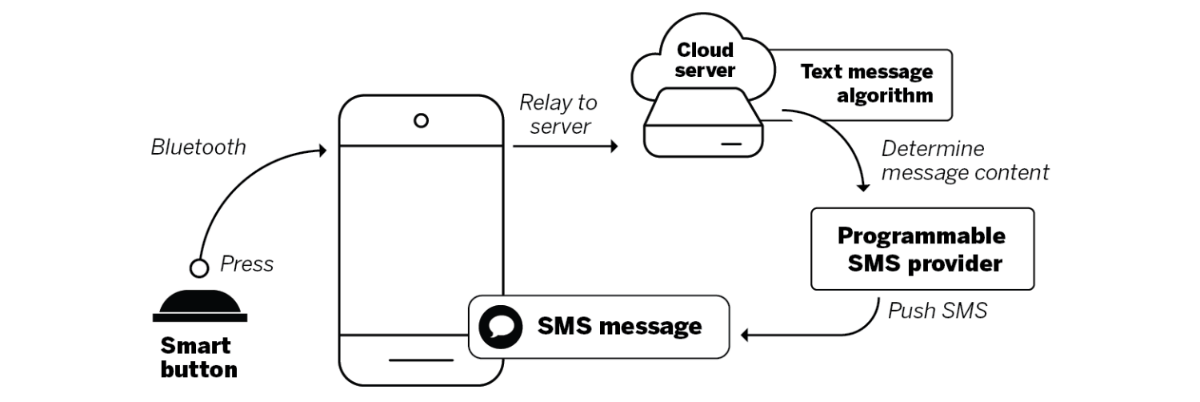
Components of the mobile health system. SMS: short message service.

The mHealth system capitalizes on Internet of Things technologies to deliver real-time SMS text messages on the basis of the time medications are taken and documented by the smart button press. The main elements of the mHealth system included a smart button and companion mobile app, a cloud-based server with a computer algorithm containing text messages, and an SMS text message platform (see Figure 1). We selected the smart button linked to a smartphone as the mHealth system, as we desired to develop an approach to measure medication taking and timing adherence without requiring users to store their medications in devices that were different from what they already used. Each of the mHealth system components is described below.

#### Smart Button and Companion Mobile App

The first element of this system is a commercially available smart button named the Stone (Pebblebee). The smart button is a Bluetooth-enabled device that can be programmed using the companion smartphone-based app called Pebblebee, hereafter referred to as the app. The smart button can be easily programmed to perform a variety of tasks, such as tracking down a lost smartphone or using the smart button to control the volume on one’s smartphone. The smart button is 0.9×0.8×0.5 inches in size, and it weighs 0.3 ounces. The battery life is listed as 1 year, and the battery is replaceable (retrieved from website). The smart button has a metal ring that allows it to be placed on a key chain. The smart button is compatible with iOS 8.3 or later and Android 5.0 or later, and it requires all phones to have Bluetooth Low Energy 4.0 (retrieved from website). The Bluetooth range is up to 150 feet, according to the manufacturer’s product information. The smartphone app interacts with the smart button. For example, when the smart button is pressed, it sends a signal via the Bluetooth connection to notify the app it was pressed. The app can be programmed to react to button presses from the smart button to perform shortcuts for tasks using a smartphone. For example, the app can be programmed so that the smart button can be used to take smartphone photos from a distance or to find a phone with the push of a button. For our purposes, we programmed the app to send an event to a cloud-based server we set up for this study using a webhook. A webhook allows real-time transfer of information to other apps running at remote locations.

#### Smartphones

Smartphones are probably the most well-known and ubiquitous smart and connected devices. A total of 3 in 4 Americans own a smartphone [19]. Smartphone capabilities such as SMS text messaging, apps, and connecting other devices to them, make smartphones potentially useful technologies to support adherence [20]. The mHealth system and use of the smart button rely on the use of a smartphone. Participants were offered study smartphones to use during the study; however, all elected to use their personal smartphones. The RA assisted participants with device setup in the clinic, including downloading and setting up the app and webhook.

#### Cloud-Based Server With a Computer Algorithm to Deliver Short Message Service Text Messages

The cloud-based server acted as an endpoint for receiving events from the smart button app via the webhook. When the cloud-based server received a notification from the app, signaling that the smart button had been pressed, both the participant and the timing of the event were used as input data by the computer algorithm to deliver subsequent SMS text messages. The computer algorithm comprised decision rules that determined the type of text message to send to participants via SMS. This association is made by the algorithm extracting the unique ID from the event received by the server and by looking up the participant who was assigned that smart button. The server processed the events according to the algorithm in Textbox 1.

**Algorithm through which the server processed events.**

If the event was received, then the algorithm determined study phase and day of study (phase 1: study days 1-14; phase 2: study days 15-45; phase 3: study days 46-52), and then subsequently the server sent an SMS text message to the participant, acknowledging they pressed the button correctly. These decision rules can be expressed as phase 1 of study={Study days 1-15}

IF participant linked with unique ID={confirmation message that system received button press notification}

If the event was received during phase 2, then the server sent a tailored SMS text message from Table N depending on the study day.

ELSE IF participant time associated with goal time={positive reinforcement message sent}

#### Programmable Short Message Service Provider

To push SMS text messages to participants, we used the SMS text platform provider Twilio, a platform as a service, which facilitates sending SMS text messages to mobile phones on behalf of an app. When the cloud-based server was ready to send an SMS text message to a participant, a request was sent to the SMS text platform, and an SMS text message was then sent to the specified participant’s mobile phone number. The SMS text platform responded with either a unique ID for the request or an error message explaining why the request failed. We assumed that receipt of a unique ID for the request implied the participant eventually received the SMS text message on the mobile phone.

#### Short Message Service Text Messages

In preparation for future intervention research, we designed 2 types of SMS text messages for this study: (1) standard and (2) tailored, which were delivered in specific phases of the study. Evidence from systematic reviews of medication adherence interventions conducted across several chronic conditions shows promise for interventions that use SMS text messaging [21-23]. The use of SMS text messaging delivered via smartphone has been shown to improve medication adherence. A total of 2 recent reviews, 1 integrative and 1 meta-analysis of randomized controlled trials, suggest that use of SMS text messages improve adherence across a variety of patient populations by approximately 17% [21,24], and therefore incorporating SMS text messages in mHealth-based interventions to improve health behavior change interventions is warranted.

##### Standard Short Message Service Text Messages

Principles of interface design indicate that providing feedback to users so they understand the technology is working is a best practice of user-centered design [25]. Accordingly, the standard text message stated “thank you for pressing the button” for participants to know that their button press was recorded (ie, the technology worked).

##### Tailored Short Message Service Text Messages

**Figure 2:**
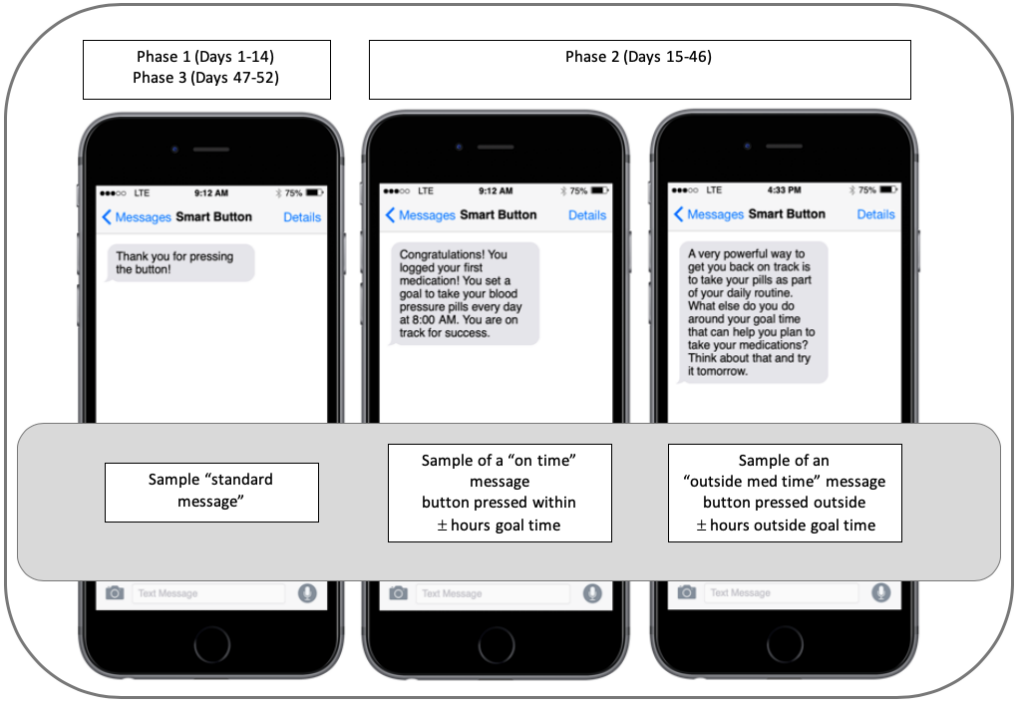
Sample short message service text messages sent across study phases.

Tailored SMS text messages were developed on the basis of systems thinking, which emphasizes habit formation using reliable systems (eg, routines) embedded in personal environments [26]. As systems thinking focuses on using established and reliable systems to support medication taking, it is an approach that moves away from “remembering.” Evidence suggests that consistent medication-taking routines support medication adherence [14], and aligning behavior with individuals’ personal environments, habits, and routines can support medication taking. Messages were tailored on the basis of whether medications were taken “On Time” or whether medications were taken “Outside Med Time.” Both types of messages were designed to draw attention to the behaviors and environments that were supporting taking medication, with the “on time” messages designed to draw attention to the environments and routines working to support taking medication and the “outside med time” messages designed to encourage thinking about processes and routines that could be changed to support taking medications on time for the next scheduled dose. SMS text feedback messages were developed to be delivered in response to smart button presses. In phase 1 and phase 3, standard SMS text messages read, “Thank you for pressing the button” on the basis of user-centered design principles that indicate users require a mechanism to determine if the technology is working as intended [25]. During phase 2, tailored SMS text messages were sent to participants on the basis of medication timing. A total of 60 SMS text messages were developed in total, with 30 “on time” messages designed to be delivered if participants took medications within ±3 hours of their regularly scheduled time, and 30 “outside goal time” messages were designed to be delivered when participants took their medications outside this dosing interval (ie, outside the ±3-hour dosing interval). Sample SMS text message content and timing of delivery is shown in Figure 2.

We set the ±3-hour window as taking a drug within 25% of the dosing interval maximizes drug bioavailability and effectiveness [27]. The SMS text messages were designed to be delivered in response to the smart button press, indicating the medication was taken. If participants pressed the button more than once daily during phase 2, the first message sent was the designated tailored medication adherence feedback based on the timing of the button press (described above), and subsequent presses on the same day triggered the same standard message participants received in phases 1 and 3.

### Feasibility Measures

#### Recruitment and Enrollment

We tracked the number of patients seen in the clinic, screened for the study, and enrolled. Reasons for not participating in the study were tracked when provided.

#### Participant Characteristics

Demographics collected at study enrollment included gender, race, ethnicity, education, marital and employment status, annual income, CKD stage, and participant self-report of coexisting medical conditions. Data were collected and entered into a study-specific Research Electronic Data Capture (REDCap) database [28].

#### Mobile Health Setup in Clinic

We tracked the number of times the mHealth set up. Setup included the ability to download the companion app, set up the webhook, press the smart button, as well as receipt of the test SMS text message that indicated the system was operating as intended.

#### Willingness to Use the System

In addition to the data tracked about participant recruitment and enrollment, we tracked instances when participants did not desire to use the mHealth system and reasons why. Dropouts and technology problems reported by participants were noted and summarized. These were identified during the scheduled RA phone calls with participants.

#### Medication Adherence

We reported medication adherence (proportion of prescribed doses taken on time) to describe adherence in this sample and better interpret the results. To measure adherence, we used an electronic medication-event monitor (EMM) pill cap that measured adherence on the basis of the number of medication bottle cap openings recorded, as measured by the Medication Event Monitoring System TrackCap (MEMSCap, AARDEX Group), expressed as a percentage. The MEMSCap contains a microelectronic circuit in the cap that registers the date and time when the cap is removed from the bottle of pills. These time stamped events are stored and can be downloaded to the web portal medAmigo [29] using a Universal Serial Bus–reader device that transfers cap data to the platform. The medAmigo is a secure cloud-based software platform, with access provided to investigators with use of the MEMSCap. Medication adherence was measured by EMM using the medication adherence scores derived from the MEMSCap and that were calculated automatically by medAmigo on the basis of the dosing history.

#### Concordance

There were 2 ways that concordance was measured. First, the number of events in which the recorded smart button events had a corresponding EMM event recorded on the same day was used. Second, we evaluated the number of these concordant events that occurred within ±5 min of one another and explored the timing in which the smart button press was activated and when the EMM device recorded a medication-taking event. The MEMSCap device was selected for the reference comparison, given its ability to measure timing adherence and evidence of its ability to estimate medication adherence [13,30-32].

#### Acceptability

Acceptability was based on qualitative feedback provided by participants. The RA documented comments made by study participants at each of 3 points of contact. The RA had 3 scheduled phone calls with participants during the study in which comments may have been documented, as well as during any points of contact when troubleshooting the mHealth system technologies may have occurred. The RA used a semistructured interview guide for phone calls. Following phase 2, the RA asked about participants’ thoughts on whether the SMS text messages were helpful to support medication taking.

### Procedure

At enrollment, all participants received study devices and in-person training by the RA on how to use the study devices. A unique ID was assigned to each button; therefore, our team could identify which participant had each button when a button was pressed. In addition, each participant received a unique study number that was used to set up the mobile app. The RA assisted participants with downloading and installing the app on their mobile phone. The app was required to run in the background of participants’ phones to detect smart button presses. The RA instructed participants to ensure the Bluetooth on their mobile device was turned on and the app was open when pressing the smart button. Next, we sent an SMS text message with information needed to configure the app to communicate with the cloud server when the smart button was pressed by the participant. This critical step allowed us to (1) confirm we had the correct mobile phone number, as the cloud server could send the participant the SMS text message, and it allowed us to (2) remove potential errors caused by transcribing setup information into the app. We could easily copy and paste the setup information into the app directly from the received SMS text message on the participant’s mobile phone. Before leaving the clinic, participants demonstrated use of the smart button, and each participant received a confirmation SMS text message indicating proper setup. To thank participants for their time in talking with the RA and using the study devices, they received an honorarium of up to US $50 in gift cards. For the purpose of this study, the RA selected 1 prescribed daily blood pressure lowering medication from participants’ prescribed medication list, and participants were instructed to keep this medication in the MEMSCap bottle. The RA instructed participants to take this medication as prescribed and press the smart button when the medication was taken. The RA asked participants to identify a date on which they would start using the study devices, and this was recorded as the study start date. In addition, the RA asked participants to identify the time when they usually took the selected medication. The date and time were recorded as the study start date, and the time was used to calculate the dosing interval. Participants were instructed to place their supply of antihypertensive medication inside the MEMSCap bottle and begin taking it from the MEMSCap bottle on the selected start date. All enrolled participants were instructed by the RA on how to use the smart button to self-track their daily medication taking. Each day, the participants were to remove their selected medication from the MEMSCap bottle at the designated time, then press the smart button. Telephone calls were made by the RA at days 15, 46, and 52 to troubleshoot technical problems, determine if participants received SMS text messages, and provide instructions on returning devices to the study team at study end.

### Data Analysis

Study data were collected and stored using REDCap electronic data capture tools, hosted at Indiana University [28]. Data were analyzed with descriptive statistics appropriate for level of measurement using IBM SPSS version 24.0 (IBM). Missing data for the smart button and EMM were coded as a failure to record data, with the exception of the days that participants reported not using the devices, and concordance analysis was adjusted appropriately. Frequency counts and percentages were used to summarize recruitment and enrollment, participant characteristics, the number of smart button press and MEMSCap events recorded, and concordance. Graphs were used to examine concordance between data acquired from self-tracking of medication adherence using the smart button and medication adherence recorded with the MEMSCap. Narrative analysis of comments made by participants was used to identify strengths and opportunities to improve the study for the future clinical trial.

## Results

### Overview

A total of 19 patients were prescreened for study eligibility in the clinic by physicians. Of these, 6 did not meet study eligibility criteria. Reasons for exclusion included the following: CKD stage >4 (n=3), no diagnosis of CKD (n=1), inability to self-manage medications (n=1), and not prescribed an antihypertensive (n=1). A total of 4 (21%) patients meeting prescreening criteria declined to participate. A total of 3 out of 4 patients provided reasons for their lack of interest in study participation. A total of 1 candidate used a flip phone, and the participant was not willing to carry another phone. The other 2 candidates were recruited for another study on day of clinic, and they were not willing to stay for recruitment discussion. Of the 3 remaining patients approached for further screening by the RA, 2 participants were willing to participate, but they were unable to stay in the clinic to obtain the study devices and be trained on setup, as they were dependent on prearranged transportation services. Of the 6 patients screened by the RA, 1 patient did not meet inclusion criteria, and the patient was excluded, as this individual was not willing to use a smartphone. A total of 5 patients were enrolled in the study, 2 participants (40%) were women, and 3 participants (60%) were men. The mean age was 52.6 years (SD 22.49; range: 20-70). Table 1 lists the self-identified health conditions of the 5 participants; 4 participants (80%) had more than 3 conditions and 2 participants (40%) had 4 or more. The 5 participants took a mean total number of 8.6 (SD 5.02) medications (range: 3-14 medications), and out of those, a mean of 2.8 (SD 1.64) were antihypertensive medications.

### Feasibility of Mobile Health System Technology Setup in the Clinic

The RA was successful in assisting all 5 participants (100%) with downloading the smart button app on their respective smartphones, setting up the webhook actions in the app, and testing the smart button device in the clinic setting. All 5 participants were able to press the smart button, and each participant received the test SMS text message while in the clinic setting, demonstrating the system was set up and operating correctly.

### Technology Challenges

A total of 2 participants reported technology problems during the study. Troubleshooting included ensuring the phone app was open and Bluetooth was turned on, as well as confirmation that the smart button was pressed when taking pills. Although each participant gave confirmation, messages were still not received by these 2 participants. A total of 1 participant texted the RA study phone to troubleshoot, as SMS text messages were not received, but the phone number the participant texted from did not match the phone number provided by the participant at study enrollment. The participant validated that only 1 mobile number was used for both voice and SMS text messages. This problem remained unresolved.

### Device Events Recorded Over Study Period

All 5 participants initiated the use of the EMM on the start day they indicated. Over the course of the 52-day study, with 5 participants, we expected to yield 260 data points (5 participants×52 days) per smart button and MEMSCap device. Over this monitored period, 36.5% (n=95) of the expected events were recorded with the smart button, and 76.2% of the expected events (n=198) were recorded with MEMSCap. There was 1 participant for whom no smart button events were recorded, although the system worked when tested in the clinic setting during enrollment.

### Measurement Concordance

**Figure 3:**
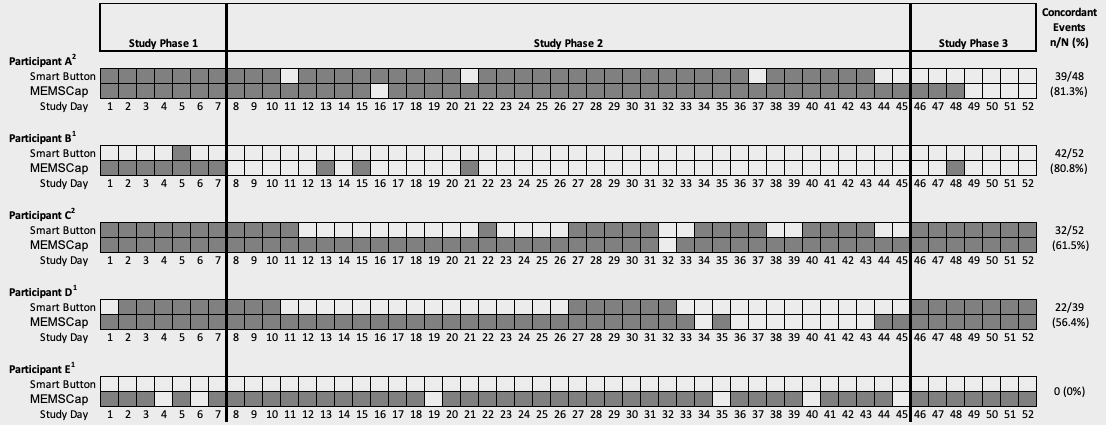
Concordance between smart button self-tracking and electronic monitoring of medication adherence. Gray boxes indicate event recorded on the device; white boxes indicate no event recorded. Type of Smartphone Operating Platform: 1Android, 2iOS; Wavy lines: participant travel, Dotted lines: refusal to use the device. Concordance determined by days participants reported using one or more of study devices. MEMS: Medication Event Monitoring.

Concordance between events recorded on both devices (n=94) was achieved on an average of 47.4% (range 0%­­-81.3%) of the time. Event recordings and concordance for each participant across the study period in which participants reported using the devices are shown in Figure 3. Among the concordant events, on average, 58.5% of the events occurred within ±5 min of one another.

We also examined these concordant events to determine which event was recorded first or if the timing of the device activation occurred at the same time. There were 34 events in which the time recorded for the smart button preceded the MEMSCap time, indicating the smart button was pressed before removing medication from the bottle. The mean difference in time between these 2 event recordings was 55 min (median 8 min; minimum: 1 min, maximum: 17 hours 6 min). In contrast, 45 events were recorded first on the MEMSCap device, with a mean time difference of 29 min (median 1 min, minimum: 1 min, maximum: 9 hours 22 min). In 15 cases, the smart button time recorded was identical to that of the MEMSCap, indicating the button was pressed at the same time medication was removed from the MEMSCap bottle. A total of 1 participant noted that during the course of the study, a pill had been removed from the MEMSCap bottle and taken, but the participant received a phone call, and then, the participant did not press the smart button until remembering to do so at a later time. A total of 1 participant ended use of the study devices on day 48 because of travel and desire not to travel with the study devices. A total of 3 participants used the devices for longer than the 52-day study period. Feasibility and Acceptability of Text Messages

Of the SMS text messages delivered, correct messages in the algorithm were sent 100% of the time. However, in 1 case, a participant traveled to another time zone, and the algorithm responded on the basis of the server time zone. Owing to the time zone difference, the medication taking was outside the goal time of the server time zone, and, as a result, the algorithm sent the appropriate feedback message, but this did not match the participant’s behavior on the basis of the new time zone. Overall, participants thought the idea of sending messages about taking medications via SMS text messages was a good idea; however, the participants shared different ideas about the timing and content of messages, as reflected in their comments below. Of the 3 participants that received the tailored SMS text messages, 2 of them found them helpful. One participant who was highly adherent to taking medications commented, “I did not find the messages helpful.” This participant suggested sending messages “when medications are taken late” (ie, outside the dosing interval) instead of sending messages when people are adherent *.* Another participant indicated the messages were all helpful but suggested to “send messages every couple of days.” This participant felt, “it was good to get encouragement when the messages came.” This same participant also indicated that the participant forgot to press the button a couple of days. Similarly, another participant noted, “I felt messages were encouraging and helpful to know I was taking my meds [ications] on track.” This participant stated that “the daily messages are helpful in motivating continued habits,” and the participant further stated that there was not “any one message that was not helpful.” Similar to the first participant comment, this participant also thought that sending a message to “prompt people who are usually late or way off” in taking medication might be helpful. The 1 participant who did not receive messages during the study commented about the helpfulness of receiving messages and indicated that “simple messages to remind to take meds would have been helpful with a timeline of when to take the medicine.”

**Table 1:** Participant characteristics.

Category		Count
**Gender**		
	Male	3
	Female	2
**Race**		
	White	4
	American Indian or Alaska Native	1
**Ethnicity**		
	Hispanic/Latino	2
	Not Hispanic/Latino	2
	Unknown	1
**Education level**		
	High school graduate	2
	Some college/no degree	1
	Bachelor’s degree	1
	Doctoral degree	1
**Marital status**		
	Never married	2
	Married	1
	Separated	1
	Widowed	1
**Employment status**		
	Full time	2
	Retired	2
	Not employed	1
**Annual income**		
	US $20,000-$30,000	1
	US $40,000-$50,000	2
	>US $100,000	1
	Prefer not to disclose	1
**Chronic kidney disease stage**		
	Stage 2	1
	Stage 3A	1
	Stage 3B	2
	Stage 4	1
**Subject-identified medical conditions**		
	High blood pressure	5
	Heart disease	3
	Arthritis	2
	Asthma	2
	Back pain	1
	Chronic obstructive pulmonary disease	1
	Diabetes	1
	Emphysema	1
	Stroke	1
	Congenital disorder	1

**Table 2:** Medication adherence acquired by electronic caps for each participant and study phase.

Participant	Overall adherence (Day 1-52), (%)	Phase 1 adherence (Day 1-14), (%)	Phase 2 adherence (Day 15-45), (%)	Phase 3 adherence (Day 46-52), (%)
A	98	100	97	100
B	22	57	6	14
C	98	100	97	100
D	95	100	91	100
E	88	86	87	100

### Medication Adherence

The average medication adherence score across the study time frame was 80.2% (range 22%-98%) when recorded using EMM. Individual participant adherence scores are shown in Table 2.

## Discussion

### Principal Findings

Existing technologies used to measure medication adherence provide data about timing and taking adherence, but they can be disruptive and intrusive in patients’ routines. This article focused on evaluating the feasibility of using a smart button to self-track medication adherence as part of an mHealth system to deliver subsequent SMS text messages on the basis of medication adherence timing. We examined the feasibility of recruiting and setting up this mHealth system in the clinic setting on participants’ own phones, patients’ willingness to use the self-tracker technology, and concordance between data acquired from the smart button and an established electronic packaging device. The main findings of this feasibility study are the lessons learned about the selected smart button and our mHealth system, which will serve to guide future studies.

### Lessons Learned and Recommendations for Future Study

We demonstrated that it is feasible to recruit participants and set up the technology in the clinic setting, which we did for the 5 individuals participating in this study. However, our study procedures that included recruiting, enrolling, and setting up technology in the same clinic appointment may have deterred some individuals from participating because of the added time to their clinic appointment. We chose this approach as a pragmatic one, but for 2 people, this approach interfered with previous transportation arrangements and precluded them from participating. Future studies should ensure study procedures are flexible to meet patient needs and maximize participation, and letting patients know in advance about potential research opportunities may be beneficial. There are benefits and challenges to participants using their own smartphones. All 5 of our participants chose to use their own phones. This was helpful at the outset, as they were familiar with the functionality of the phone. However, as Ling [33] points out, having expectations that mobile phones all provide the same level of access and functionality in designing and delivering mHealth interventions is often taken for granted. While troubleshooting technological problems, our team discovered that individual privacy settings were interfering with delivery of messages. A total of 2 iPhone users had a setting that if the SMS text message sender was unknown, the messages would be automatically deleted. On the basis of this finding, we recommend that in future research, phone numbers associated with the study be added to participant phone contact lists at study outset so that participants receive study messages and phone calls. One of the greatest challenges we encountered in our study was that the smart button only worked when the companion app was running in the background on the phone. Despite knowing this in advance and teaching our participants to keep the app open, there is a high likelihood that the app was closed, and it is the reason why smart button presses were not received. Smart button use for self-tracking medication adherence would be more useful if participants did not need to worry about the companion app. Moving forward with future research, we recommend exploring if other smart buttons on the market can operate without requiring the user to take this extra step or designing new systems that overcome this obstacle. We also learned that time zone changes posed challenges for our mHealth system, and time zone changes posed challenges for measuring medication adherence. As the determination of medication adherence is based on date and time, travel to other time zones can interfere with a regular schedule of medication taking. The smart button is connected to the phone, so the time zone of the user is the appropriate time zone, but if the server uses the time zone to send message algorithms, such as the one our team designed, then the messages may not appropriately match the medication-taking behavior. We found a study reported in the literature that encountered a similar difficulty with mHealth technology and medication adherence, and those researchers changed the programming code to ensure medication time was based on the mobile phone users’ local time rather than the server or research teams’ time zone [34]. We believe this a potential problem for all types of electronic measures of adherence, especially if the measurement device does not have the capability of sensing and responding to geographical changes. For example, the EMM MEMSCap device we used does not sense time zone changes autonomously; therefore, this is something that users would need to report to investigators to more accurately measure adherence if participants travel while having their medication taking monitored. On the basis of these findings, future investigations should ensure the programming of the mHealth system can respond appropriately to time zone changes. Investigators may wish to have patients keep a log of any time zone changes that occur during the course of their medication monitoring in medication adherence studies, including in studies using existing electronic medication monitoring devices. We also learned that the technology may not work outside of the United States; therefore, this is an important consideration for planning future research. Future research should explore the ideal conditions under which the smart button has utility both from a measurement and intervention perspective. In future research, there is a need to consider individual user characteristics, including gender, race, ethnicity, income, number and types of medications taken, and different chronic conditions, to determine if using a smart button self-tracker and mHealth system is feasible and then subsequently determine if using a smart button self-tracker and mHealth system can improve medication adherence. Future research will also need to further evaluate the content of tailored SMS text messages for content and face validity congruent with systems thinking and determine the best timing for delivering messages. Engaging patients in the participatory codesign of these messages may be a salient opportunity and best practice approach to engage the target end user [35,36].

### Limitations

This is a small feasibility study focused on evaluating the technology components of the mHealth system and the ability to use a smart button to self-track medication adherence in the field. The sample size is small, but it was purposefully chosen to test feasibility of the smart button and mHealth system to operate according to plan in the patient home environment. Although the sample size limits generalizability, the study was useful in identifying opportunities to improve future iterations of the mHealth system components. Although we assessed medication adherence using an EMM bottle device and asked about self-reported adherence to devices, we do not have baseline medication adherence data for participants. Self-reported adherence, although likely to overestimate adherence, should be evaluated in future studies at baseline. Another limitation was that we relied on the SMS text message data to determine if the smart button was pressed. Participants reported pressing the button, yet no events were received on the server, and therefore no SMS messages were generated. Whether or not the participants pressed the button was not objectively evaluated in the study procedures; therefore, we do not have data on whether participants actually pressed the button. One of the challenges in medication adherence research is that there is no gold standard measure of adherence. The wide variability in measurement methods and ways of acquiring information on medication taking (self-report, indirect, and direct) are challenges in conducting this research. The smart button provided a self-report measure of medication taking, and we used the EMM bottle device to provide an indirect objective measure of adherence. We recognize that both of these methods come with limitations, which is why we used the concordance measure to make comparisons, but nonetheless, without direct observation, both of these approaches provide only an estimate of adherence. As the smart button is a self-report measurement approach, it is limited to patients’ willingness to actually report their medication taking. However, our smart button approach is an innovative way to self-report medication adherence, as it allows for real-time self-report of medication adherence and does not rely on recalling if medications were taken, which is the basis for most self-report measures of adherence [6]. Among self-report measures that rely on recall, adherence is often overestimated by approximately 30% [37]. The smart button component of our mHealth system provides a novel self-report approach to measuring medication adherence, as it provides time and date data that are lacking from other common self-report methods for measuring adherence.

### Conclusions

We demonstrated that it is feasible for participants recruited from the clinic setting to use a smart button device to self-track medication taking, although the selected device may not reliably work across smartphone operating systems and in participant home environments. Adherence was relatively high in this sample, although corresponding smart button presses were not consistently recorded, demonstrating poor concordance. We believe the discrepancy lies in the selected smart button technology and not in the ability of patients to self-track their medication taking, although this requires further study. Although there are some limitations to the use of the specific smart button used in this study, we were able to identify opportunities to improve the system to support testing in future studies.

## Abbreviations

CKD: chronic kidney disease
EMM: electronic medication-event monitor
MEMSCap: Medication Event Monitoring System TrackCap
mHealth: mobile health
RA: research assistant
REDCap: Research Electronic Data Capture
SMS: short message service
